# Target network differences between western drugs and Chinese herbal ingredients in treating cardiovascular disease

**DOI:** 10.1186/1471-2105-15-S4-S3

**Published:** 2014-03-19

**Authors:** Peng Fu, Linlin Yang, Yi Sun, Li Ye, Zhiwei Cao, Kailin Tang

**Affiliations:** 1 State Key Laboratory of Bioreactor Engineering, East China University of Science & Technology, Shanghai 200237, China; 2 Shanghai Center for Bioinformation Technology, Shanghai 201203, China; 3 School of Life Science and Technology, Tongji University, Shanghai 200092, China

## Abstract

**Background:**

Western drugs have achieved great successes in CVDs treatment. However, they may lead to some side effects and drug resistance. On the other hand, more and more studies found that Traditional Chinese herbs have efficient therapeutic effects for CVDs, while their therapeutic mechanism is still not very clear. It may be a good view towards molecules, targets and network to decipher whether difference exists between anti-CVD western drugs and Chinese herbal ingredients.

**Results:**

Anti-CVD western drugs and Chinese herbal ingredients, as well as their targets were thoroughly collected in this work. The similarities and the differences between the herbal ingredients and the western drugs were deeply explored based on three target-based perspectives including biochemical property, regulated pathway and disease network. The biological function of herbal ingredients' targets is more complex than that of the western drugs' targets. The signal transduction and immune system associated signaling pathways, apoptosis associated pathways may be the most important pathway for herbal ingredients, however the western drugs incline to regulate vascular smooth muscle contraction associated pathways. Chinese herbal ingredients prefer to regulate the downstream proteins of apoptosis associated pathway; while the western drugs incline to regulate the upstream proteins of VECC (Vascular Epidermal Cells Contraction) related pathways.

**Conclusion:**

In summary, the characteristics identified in this study would be valuable for designing new network-based multi-target CVD drugs or vaccine adjuvants.

## Background

According to the statistics of The World Health Organization (WHO), CVDs are the world's largest killers of human health, since these disorders lead to 17.1 million deaths per year and the death number is still rising [1]. Cardiovascular diseases (CVDs) is the general term that describes a number of circulatory system diseases, which mainly include ischemic heart disease, hypertension, cardiac arrhythmias, stroke, myocardial infarction, coronary artery disease, hypertrophic cardiomyopathy, hyperlipidemia, etc [2,3]. Both genetic abnormalities and environmental factors play important roles in CVDs development. Their pathological mechanisms often refer to many complex physiological processes, such as inflammation [3], apoptosis [4,5], oxidative stress [6], and lipid metabolism [7].

There have been various anti-CVD drugs approved by US FDA. One of the most-used examples is the statins. They have reduced the mortality and morbidity from atherosclerotic heart disease by about 30%, but the remaining 70% CVDs still need novel therapies [8]. In recent years, with the development of genomics [9] and proteomics [10], a large number of associated genomic and gene regulation data make it possible to develop new drugs for CVDs. Bezafibrate [11], Lisinopril [12], Quinapril [13] and so on, have been developed as new drugs of CVDs. Although western drugs have achieved great successes in CVDs treatment, they may lead to serious side effects [14] and drug resistance [15].

On the other hand, more and more studies found that Chinese herbs have efficient therapeutic effect for CVDs, such as Danshen Pian [16,17], Yin Xing Ye Pian [16,18], Shengmai Yin [16] and Xinning Pian [16]. For instance, Danshen has a range of potentially beneficial effects, including causing coronary vasodilatation, suppressing the formation of thromboxane, and inhibiting platelet adhesion and aggregation [19]. *G. biloba *extract (GBE) was found to decrease capillary permeability, inhibit platelet-activating factors, and decrease vascular resistance [20]. Herbal ingredients have been expected as a potential drug like database [21]. In addition, increased research has been carried out in search of new adjuvant candidates from traditional Chinese medicinal herbs [22,23]. Although great promise has been shown for Chinese herbs, their therapeutic mechanism is still not very clear.

Before demystify the mechanism of anti-CVD Chinese herbs, researchers want to know whether there is any difference between herbal ingredients and western drugs? It may be a good view towards molecules and their targets to decipher these questions. In this study, *herbal ingredients *(active compounds in herb which have been reported with anti-CVD effects) and *western drugs *(FDA-approved drugs with anti-CVD effectiveness) have been comprehensively collected. Their corresponding target proteins were retrieved from DrugBank [24] and HIT [25]. Then, the similarities and the differences of molecular mechanism between those *herbal ingredients *and *western drugs *were probed from 3 target-based perspectives: biochemical property, regulated pathway, and disease related network.

## Methods and materials

### Data preparation

#### Molecules, targets and related diseases

*Western drugs *whose first letter of ATC code is "C" and their associated targets were downloaded from DrugBank. The keywords such as: "Hypertension", "Hyperlipidemia", "Coronary heart disease", "Stroke", "Myocardial infarction", "Angina", "Atherosclerosis", "Cardiomyopathy", "Heart failure" and "Thrombosis" have been used to search anti-CVD herbs in Chinese Pharmacopoeia [16]. The ingredients of anti-CVD herbs and their corresponding targets were extracted from HIT [25]. Two kinds of ingredients' targets have been collected from HIT: direct targets and indirect targets.

Targets and associated diseases were collected from Therapeutic Target Database (TTD) [26]. As targets are under different stages of study, they have been classified into three categories in TTD: Successful targets, Clinical trial targets and Research targets.

#### Protein biochemical family, structure domain and cell location

Protein Biochemical family data was downloaded from UniProtKB [27]. Protein structure domain data was obtained from Pfam [28]. Protein cell location data was collected from The Gene Ontology (GO) [29]. GO provides an ontology of defined terms representing gene product properties, cell location information for the targets was extracted from Cellular component item.

#### Protein transcription factor

TRANSFAC database records eukaryotic transcription regulating DNA sequence elements and the transcription factors binding to and acting through them [30]. Information of transcription factor was retrieved from TRANSFAC, 618 transcription factors have been included in TRANSFAC.

### Network construction and analysis

#### Construction of target-pathway network and compound-pathway network

Firstly, target proteins of *herbal ingredients *and *western drugs *were mapped onto KEGG pathways [31] respectively. Targets were considered to participate in a specific pathway if they appear in the pathway. Through regulating those targets, *western drugs *or *herbal ingredients *act on pathways. A bipartite graph was constructed by linked pathways and targets, which represents the association of targets and pathways [32]. In the same way, the compound (a compound means an herbal ingredient or a western drug)-pathway network was generated.

#### Degree distribution of network

In network, the number of edges linked to a node was defined as degree. The degree distribution *f(x) *of network is the frequency of nodes with degree x. It is reported that degree distribution of the majority of real world networks especially biological networks obey power law. In another word, the majority of nodes in network would only influence few nodes, while few nodes would affect a lot of nodes and play important roles in whole network [32,33].

#### Pathway enrichment for targets of *herbal ingredients *and *western drugs*

Pathway enrichment analysis [34] has been applied to query whether a compound regulate a pathway by chance or not. Fisher's exact test was used. A significance level with a Pvalue less than 0.01 means the compound would regulate the pathway at a high probability [32].

#### Construction of disease network

In previous reported studies, inflammation, apoptosis and Ca^2 + ^channel caused vascular epidermal cells contraction disorder were the main pathogenesis for cardiovascular diseases. Firstly, two CVDs local networks were constructed: the *CVDs-Apoptosis-Network *and the *CVDs-VECC *(vascular epidermal cells contraction)*-Network*. Secondly, the *whole-CVDs-network *was built which integrates CVDs associated pathways in KEGG, BioCarta [35] and Therapeutically Relevant Multiple Pathways (TRMP) [36].

## Results and discussions

### Targets of *herbal ingredients *and *western drugs*

184 FDA-approved anti-CVD *western drugs *as well as their 204 protein targets were collected from DrugBank. 40 anti-CVD herbs were collected in the Chinese Pharmacopoeia as well. For these anti-CVD herbs, 172 herbal ingredients and 862 protein targets were identified from the HIT, among which 118 were direct targets.

Targets of *herbal ingredients *and *western drugs *were mapped to TTD [37] (Table 1). Targets in TTD have been mainly grouped into three categories: Successful targets, Clinical trial targets and Research targets. Among the collected *western drug'*s targets, 81(39.7%) are successful targets, 15(7.4%) are clinical trial targets, 29(14.2%) are research targets and 2(1.7%) are discontinued targets. For the direct targets of *herbal ingredients*, 32(27.1%) are successful targets, 10(8.5%) are clinical trial targets, 32(27.1%) are research targets and 3(2.5%) are discontinued targets. Additionally, among all targets (including direct and indirect targets) of *herbal ingredients*, 84(9.7%) are successful targets, 88(10.2%) are clinical trial targets, 180(20.9%) are research targets and 14(1.6%) are discontinued targets. Totally, 62.3% targets of *western drugs*, 65.3% of direct targets *herbal ingredients *and 41.5% all targets of *herbal ingredients *are recorded in TTD. *Herbal ingredients*' direct targets were more similar to *western drugs*' targets than all targets of *herbal ingredients*. To better compare *herbal ingredients *and *western drugs*, the bias of their target number and target type should be excluded. Only direct targets of *herbal ingredients *were used in the following study.

**Table 1:** Protein targets of *herbal ingredients *and *western drugs *in TTD.

Class	*western drugs*-targets(204)		*herbal ingredients *-direct targets(118)		*herbal ingredients *-all targets(862)	
	
	number of targets	Percentage	number of targets	Percentage	number of targets	Percentage
Successful targets	81	39.7%	32	27.1%	84	9.7%
Clinical trial targets	15	7.4%	10	8.5%	88	10.2%
Research targets	29	14.2%	32	27.1%	180	20.9%
Discontinued targets	2	1.7%	3	2.5%	14	1.6%
Sum of targets in TTD	127	62.3%	77	65.3%	358	41.5%

*D/t values *(the average value for the number of associated diseases for each target) were calculated for *herbal ingredients' *and *western drugs' *targets (Table 2). *Herbal ingredients *showed higher *d/t value *than *western drugs *in each category. The average number of *herbal ingredients' *targets associated diseases is higher than that of western ones. It implies that *herbal ingredients*' targets may associate with more diseases than the targets of *western drugs*.

**Table 2:** Drug targets related diseases from TTD.

Type of targets		Number of diseases	d/t
	Successful target(81)	221	2.73
*western drugs*	Clinical trial target(15)	45	3.0
	Research target(29)	56	1.93
	Successful target(32)	160	5.0
*herbal ingredients*	Clinical trial target(10)	39	3.9
	Research target(32)	76	2.38

(d/t represents the average value for the number of associated diseases for each target)

**Figure 1:**
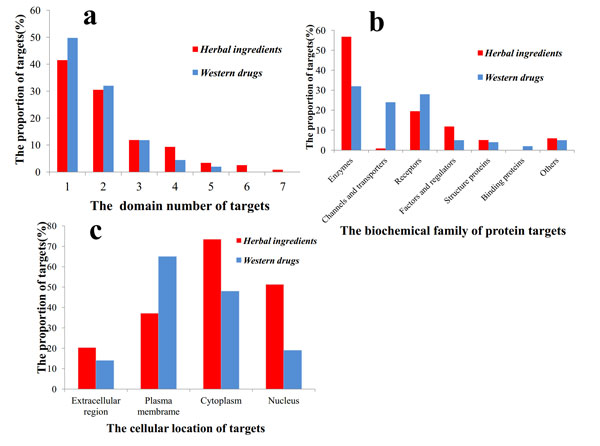
**a. the domain number of *herbal ingredients *and *western drugs *targets**. b. Distribution of main protein biochemical family of *herbal ingredients *and *western drugs*. c. Distribution of subcellular location of targets.

To some extent, the number of targets associated-diseases depends on the complexity of the biological function of the target. Since the domain types of protein targets were often used to measure the biological function complexity, distribution of the number of structure domain for targets of *western drugs *and *herbal ingredients *were obtained by Pfam (Figure 1a). The numbers of structure domain of targets from *herbal ingredients *tends to be more than those of *western drugs*. It indicates that the biological function of the *herbal ingredients' *targets is more complex than that of the *western drugs*. This is also consistent with the observation from the *d/t value *analysis. In another word, *herbal ingredients' *targets would link to more diseases and prefer to participate in more complicated biological functions.

As shown in Additional file 1, figure S1, 27% of western drugs bind different subtypes of the same type of protein targets such as inhibitors of calcium ion channel etc. The other 28% of western drugs regulate different targets in the same biological process. That is to say, besides one-target-drugs, most targets of multi-targets western drugs either belong to the same protein type or participate in the same biological process. This might hint why western drugs are often used for a certain disorder.

Protein targets were classified into certain families: enzymes, channels and transporters, receptors, factors and regulators, structure proteins, binding proteins and others [38]. As seen from Figure 1b, targets of *herbal ingredients *were enriched in enzymes, factors and regulators. *Western drugs*' targets were enriched in receptors, channels and transporters. To some extent, the biochemical family of proteins depends on the cellular location of the proteins. The cellular locations of targets from *herbal ingredients *and *western drugs *were extracted from GO (Figure 1c). Targets of *herbal ingredients *preferred to locate in cytoplasm and nucleus, while western drugs preferred to locate in plasma membrane. As we know, enzymes, factors and regulators often locate in cytoplasm and nucleus, channels and transporters, receptors usually locate in plasma membrane. The results in cellular location accorded with the bio-chemical family analysis. Therefore, *herbal ingredients *may prefer to target enzymes, factors and regulators in cytoplasm and nucleus, while *western drugs *may prefer to target channels and transporters, receptors in plasma membrane.

11 targets of 118 *herbal ingredients' *direct targets were TFs (transcript factor), while only 10 out of 204 western drugs targets were TFs (Table 3). Fisher's exact test was used to quantitatively measure which kind of targets is more enriched with TFs. The P-value of *herbal ingredients *is less than 0.01, however *western drugs*' is greater than 0.05. It implies that *herbal ingredients *prefer to target TFs than *western drugs*.

**Table 3:** Transcription factor distribution for targets of western drug and *herbal ingredients*

	Uniprot-id	Factor name	Num for Transcriptional genes
	P10275	AR	3
	P05412	c-Jun	19
	P04150	GR-alpha	5
*Western drugs*	P08235	MR	0
	P10827	T3R-alpha	4
	P10828	T3R-beta1	5
	P35869	AhR	7
	Q07869	PPAR-alpha	0
	Q03181	PPAR-beta	1
	P51843	DAX1	0
	P05412	c-Jun	19
	P03372	ER-alpha	8
	Q04206	RelA	12
	Q00613	HSF1 (long/low)	1
*herbal ingredients*	Q09472	p300	15
	P40763	STAT3	3
	P35869	AhR	7
	Q07869	PPAR-alpha	0
	Q03181	PPAR-beta	1
	O75469	PXR-1/SXR	0
	Q92731	ER-beta	1

(Fisher's exact test P-value, *herbal ingredients*: 0.00108, *Western drug*: 0.14684)

As summarized from the above protein family analysis, cellular location analysis, and the TF enrichment analysis, the targets of *western drugs *are enriched in channels and transporters, receptors, and they tend to locate on the plasma membrane, which agrees with the common sense about western drug discovery. On the other hand, the targets of *herbal ingredients *are enriched in enzymes, factors and regulators, and they prefer to locate in cytoplasm and nucleus. This suggests that *herbal ingredients *may provide new insights for CVDs therapy. In addition, as the *herbal ingredients' *targets locate in cytoplasm and nucleus, this may meet difficulties in exploring *herbal ingredients' *mechanism.

### Pathways analysis

**Figure 2:**
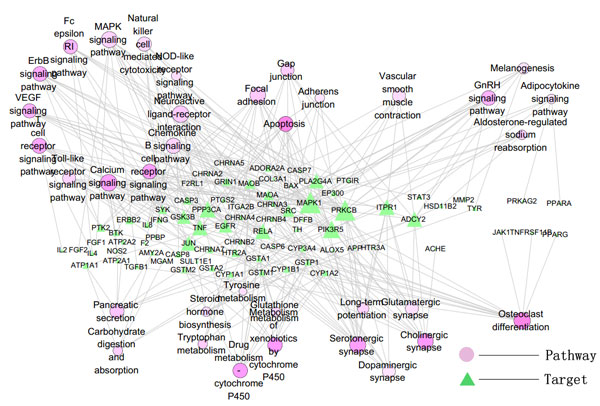
**The target-pathway network of *herbal ingredients *(The gradient of color and size for the pathway-nodes changes according to the enrichment (P-value) and degree: light red for pathways with big P-value, while deep red for pathways with small P-value; big node for pathways with high degree, while small node with low degree)**.

To get an overall view of the interplays between *herbal ingredients*/*western drugs *and KEGG PATHWAY, we mapped all the targets of *herbal ingredients *and *western drugs *onto the KEGG PATHWAY [31] respectively. It is found that they are enriched in 65(*herbal ingredients*) and 35(*western drugs*) pathways (P-value < 0.01). However, no matter for *herbal ingredients' *or *western drugs *targets, at least 33% pathways of the enriched pathways was Disease pathway (Additional file 2, figure S2). We next mapped *herbal ingredients *and *western drugs *targets onto the basic KEGG PATHWAY (without the disease pathway in KEGG PATHWAY) respectively and found that they enriched in 35(*herbal ingredients*) and 25(*western drugs*) basic KEGG pathways. Target-pathway network of *herbal ingredients *and *western drugs *were illustrated in Figure 2, 3. In *herbal ingredients target-pathway network*, signal transduction pathways and immune system associated signaling pathways, apoptosis associated pathways interacted with quite plenty of targets, and these pathways also showed high significance enrichment. In *western drugs target-pathway network*, cardiac and vascular smooth muscle contraction associated pathways interacted with many targets.

**Figure 3:**
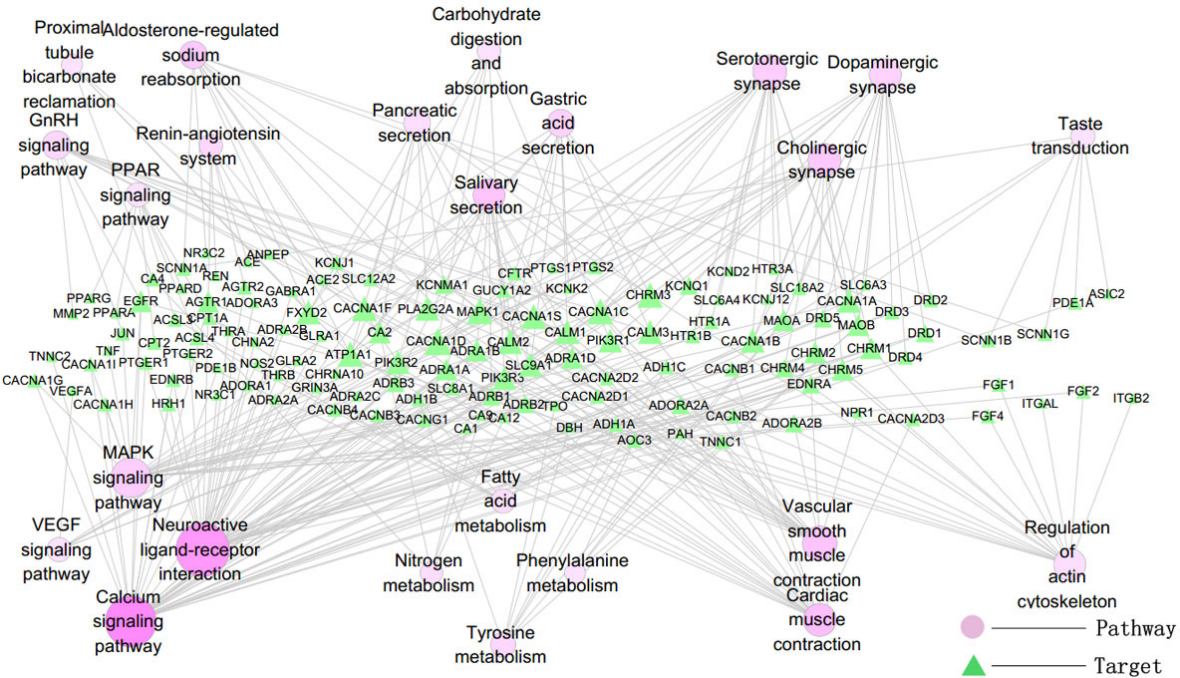
**The target-pathway network of *western drugs *(The gradient of color and size for the pathway-nodes changes according to the enrichment (P-value) and degree: light red for pathways with big P-value, while deep red for pathways with small P-value; big node for pathways with high degree, while small node with low degree)**.

**Figure 4:**
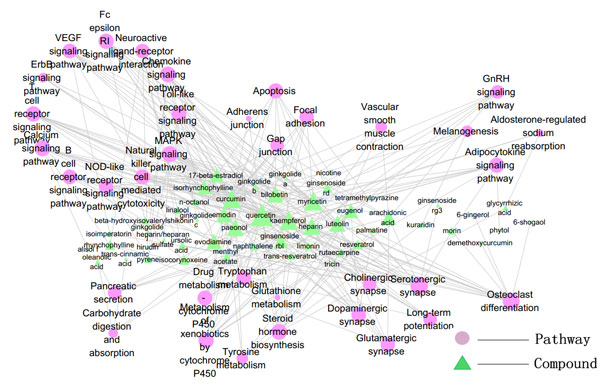
**The compound-pathway network of *herbal ingredients *(The gradient of color and size for the pathway-nodes changes according to the enrichment (P-value) and degree: light red for pathways with big P-value, while deep red for pathways with small P-value; big node for pathways with high degree, while small node with low degree)**.

**Figure 5:**
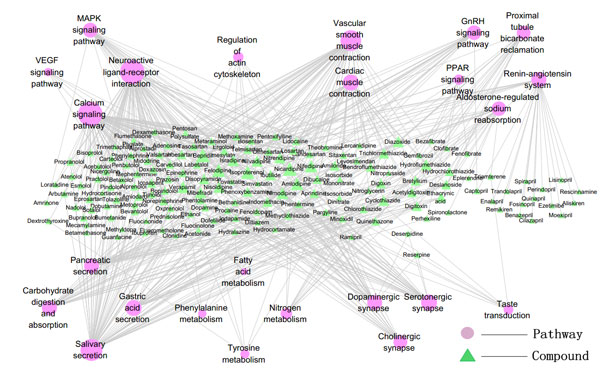
**The compound-pathway network of *western drugs *(The gradient of color and size for the pathway-nodes changes according to the enrichment (P-value) and degree: light red for pathways with big P-value, while deep red for pathways with small P-value; big node for pathways with high degree, while small node with low degree)**.

Compound-pathway network was constructed, where a compound and a pathway were linked each other if one target of the compound was included in that pathway (Figure 4, 5). The signal transduction and immune system associated signaling pathway, apoptosis associated pathways have a higher degree than others in herbal ingredient-pathway network. Vascular smooth muscle contraction associated pathways showed high degree in drug-pathway network for western drugs.

It can be inferred from both target-pathway and compound-pathway analysis that signal transduction and immune system associated signaling pathways, apoptosis associated pathways are always the most important pathway of *herbal ingredients*, while for the western drugs, the most important pathways are cardiac and vascular smooth muscle contraction associated pathways. These herbal ingredients can be exerted along with the vaccine to elicit a faster and stronger immune response [22].

### Disease network

**Figure 6:**
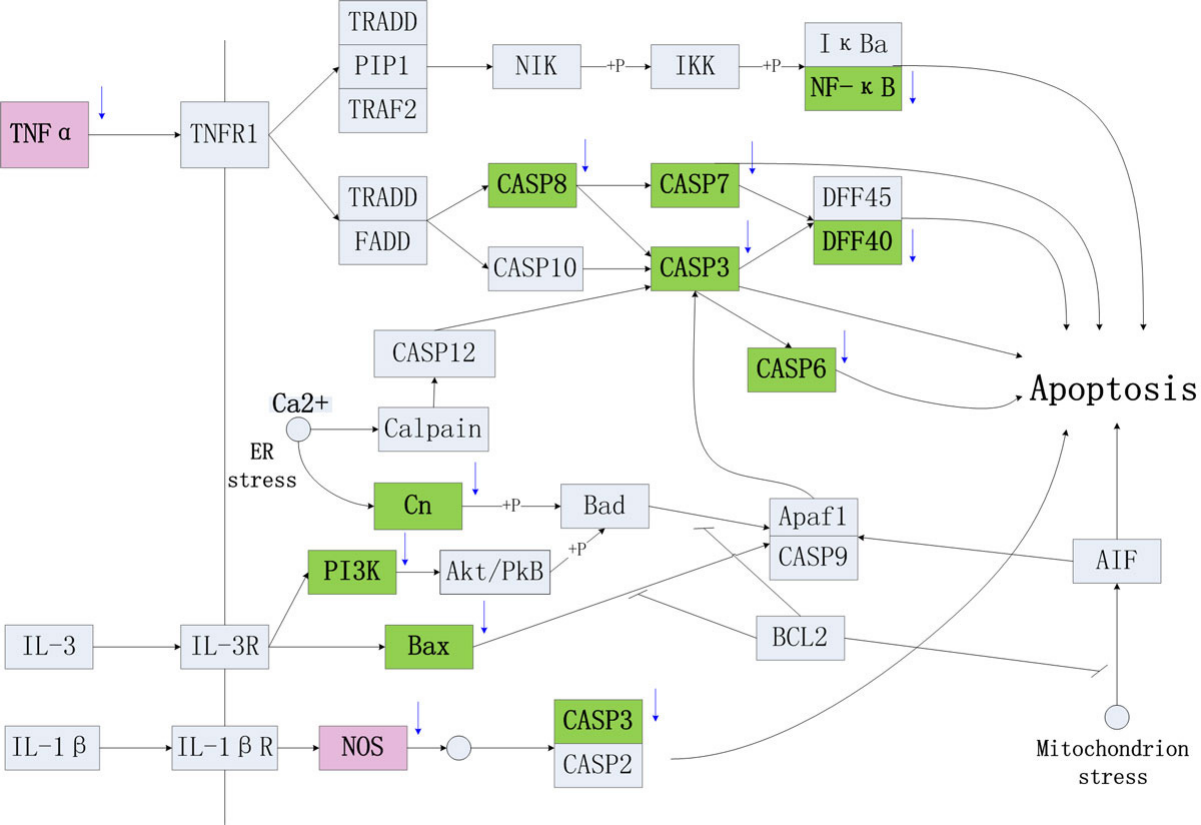
**The *CVDs-Apoptosis-Network *(Different colors represent Different type of targets: the green is the targets of *herbal ingredients*, and the light red is common targets for western drugs and herbal ingredients**. The arrow near the rectangle represents the activities of compounds for the protein: up means activation, down means inhibition).

In order to further pathway analysis, we constructed *CVDs-Apoptosis-Network *and *CVDs-VECC *(Vascular Epidermal Cells Contraction)*-Network *and *CVDs-Whole-Network*. In previous studies, cellular apoptosis plays an important role in the development of some CVDs [39], inhibiting apoptosis was an effective way to prevent and control heart failure [5]. The *CVDs-Apoptosis-Network *was constructed (Figure 6) by integrating known apoptosis associated pathways in the process of CVDs development. There were many targets of *herbal ingredients *participated in the network, but only two targets of *western drugs *acted on it. The targets participated in *CVDs-Apoptosis-Network *further indicate that *herbal ingredients *preferred to cure CVDs through regulating cellular apoptosis.

**Figure 7:**
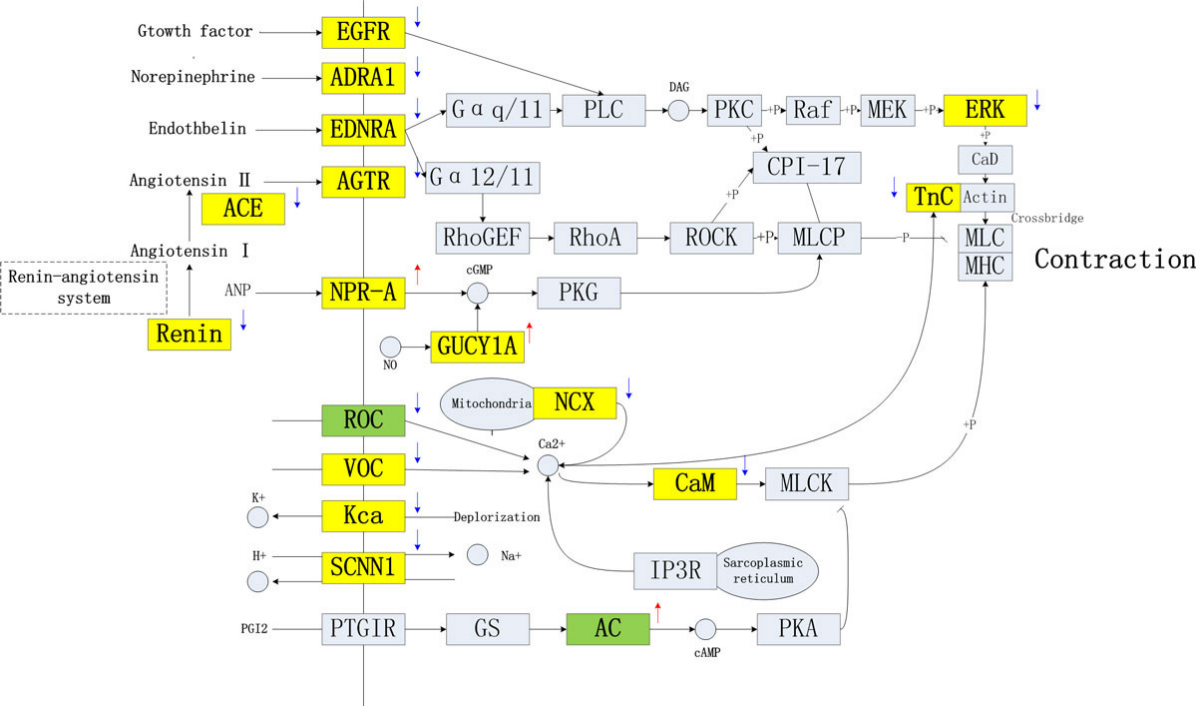
**The *CVDs-VECC *(Vascular Endothelial Cell Contraction)*-Network *(Different colors represent Different type of targets: the yellow is the targets of *western drugs*, and the green is the targets of *herbal ingredients***. The arrow near the rectangle represents the activities of compounds for the protein: up means activation, down means inhibition).

Vascular Endothelial Cell Contraction (VECC) was one of the factors for the development of some CVDs, and some drugs had been reported to cure hypertension by regulating vascular endothelial cell contraction associated pathways [40]. Thus, a *CVDs-VECC-Network *(Figure 7) was constructed by integrating VECC associated pathways in the process of CVDs development. Many of the targets of western drugs present in the network, while only two targets of *herbal ingredients *presented. This agreed with the hint from former pathway analysis that *western drugs *preferred to target cardiac and vascular smooth muscle contraction associated pathways.

**Figure 8:**
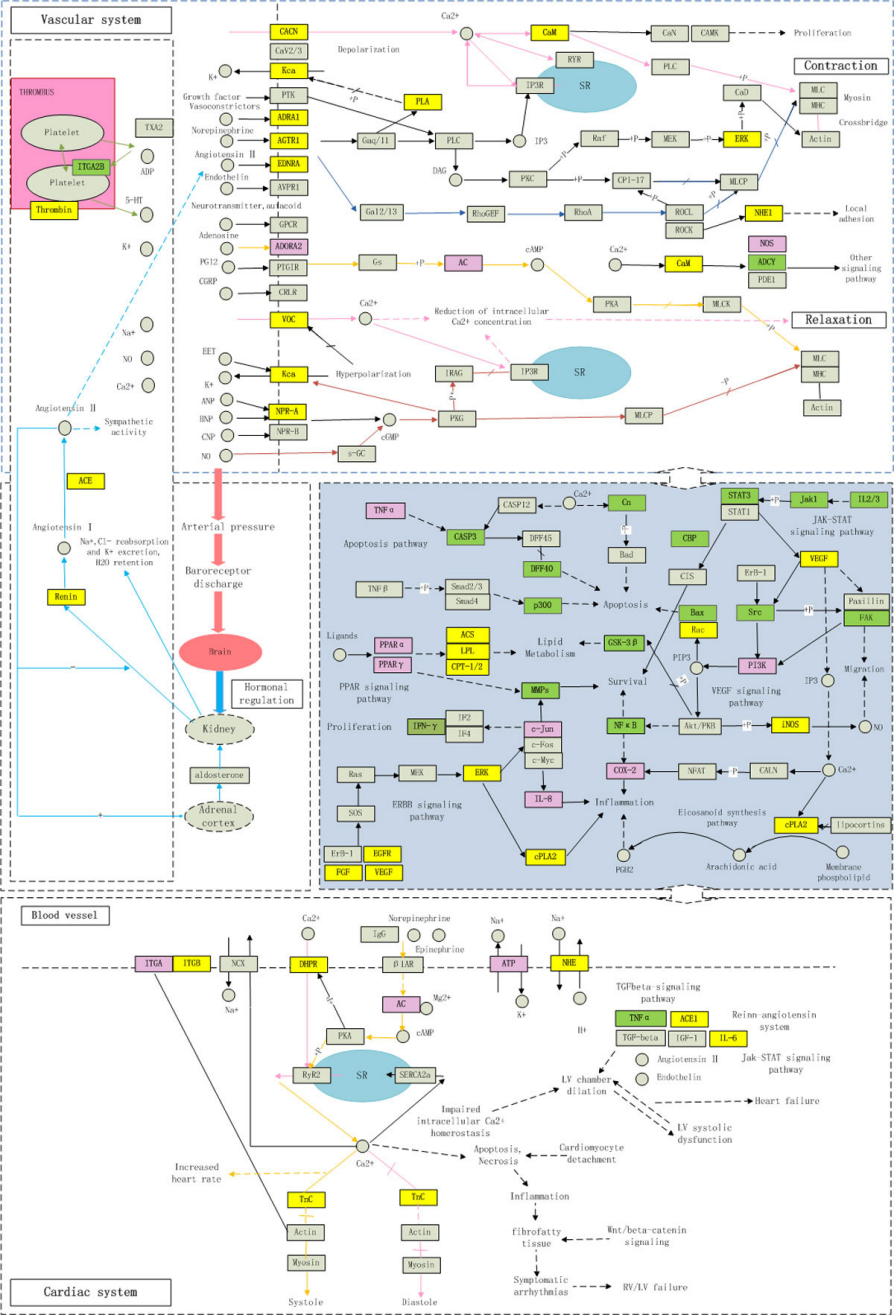
**The *CVDs-Whole-Network *(Different colors represent Different type of targets: the yellow is the targets of *western drugs*, the green is the targets of *herbal ingredients*, and the light red is common targets of *western drugs *and *herbal ingredients***. The arrow near the rectangle represents the activities of drugs or *herbal ingredients *for the protein: up means activation, down means inhibition).

The *CVDs-Whole-Network *was constructed by integrating CVDs associated pathways included in KEGG and Therapeutically Relevant Multiple Pathways (TRMP) Database (Figure 8). *Western drugs *trend to target blood proteins and membrane proteins, while *herbal ingredients *only targeted one blood protein and most of its targets located inside the cell.

The *CVDs-Whole-Network *and the two local networks provide an overview that *western drugs *prefer to target the upstream of CVDs, while the *herbal ingredients *prefer to modulate the downstream of CVDs.

### Adjuvant discussion

Use of adjuvant in the vaccine preparation is a long standing practice. Despite major advances in vaccine adjuvants, vaccines seem to depend on aluminium salts. However, these adjuvants would lead to serious adverse effects [41]. Herbal ingredients as immuno-modulator are paving its way as a safe alternative.

From the results of pathway enrichment, *herbal ingredient*s' direct target significantly enriched in seven immune system pathways, while no immune system pathway was enriched by *western drugs' *targets. For example, curcumin (the extractive of radix curcumae) would not only regulate interleukin 1(IL1), interleukin 6(IL6), interleukin 8(IL8), interferon-γ(IFN-γ) and Transforming Growth Factor beta(TGF-β) in T and B cell, but also target some immune proteins such as, Toll-like receptor 4(TLR4), CD80 and CD28. Styrene (the extractive of storax) can affect IL4, IL5, IL13 and TGF-β expression. Resveratrol (the extractive of polygonum cuspidatum) can regulate IL1, IL6, IL8, IL10, IFN-γ, TGF-β, CD80 and CD28. What's more, ginseng saponins have adjuvant effects on the specific immune responses, which due to their can promote IL1 production by pertoneal macrophages, stimulate secretion of cytokine such as IL2, IL4, IL6, IL8, IL10, IFN-γ and TNF-α, regulate TLR4 to produce proinflammatory cytokines, and affect TGF-β to enhance lymphocyte proliferation [42-45]. Therefore, those herbal ingredients could regulate one or several kinds of cytokine such as IL1, IL4, IL6, IL8, IL10, IFN-γ, TGF-β and immune related protein TLR4 have the potential to promote both humoral and cellular immune responses. Some ingredients of anti-CVD herbs might be the candidate adjuvant.

## Conclusion

*Herbal ingredients *and *western drugs*, as well as their targets were thoroughly collected in this work. The similarities and the differences between *herbal ingredients *and *western drugs *were deeply explored. From biochemical property analysis, the biological function of *herbal ingredients' *targets is more complex than that of the targets of *western drugs*. *Herbal ingredients *tend to target enzymes, factors, regu-lators protein in cytoplasm and nucleus, while *western drugs *lean towards targeting the protein of channels and transporters, receptors in plasma membrane. From pathway analysis, the signal transduction and immune system associated signaling pathways, apoptosis associated pathways are always the most important pathway for *herbal ingredients*, however *western drugs *incline to regulate vascular smooth muscle contraction associated pathways. From disease network analysis, *herbal ingredients *prefer to regulate the downstream proteins of apoptosis associated pathway, while the *western drugs *incline to regulate the upstream proteins of VECC related pathways.

According to some statistics, nearly one-third of the top-selling drugs in the world are nature products or their derivatives, and nature products are the most consistently successful source of drug leads [46,47]. Herbal ingredients may provide some new clues to drugs development for CVDs. Herbal ingredients may also have the potential to be used in design of new vaccines.

## Authors' contributions

PF and LY collected data and performed the computational data analysis. ZWC and KLT conceived of the study, participated in its design and coordination and revised the manuscript. LLY and YS participated in the design and helped to review the manuscript. All authors read and approved the final manuscript.

## Competing interests

The authors declare that they have no competing interests.

## Supplementary Material

Additional file 1 **Figure S1 The distribution of drug type for western drugs**. Click here for file

Additional file 2 **Figure S2 The proportional distribution of enriched KEGG pathway**. Click here for file

## Declarations

The publication of this work was funded by Ministry of Health (2012ZX10005001-008).

This article has been published as part of BMC Bioinformatics Volume 15 Supplement 4, 2014: Selected articles on Computational Vaccinology 2013. The full contents of the supplement are available online at http://www.biomedcentral.com/bmcbioinformatics/supplements/15/S4.

## References

[B1] Cartwright EJ Oceandy D Austin C Neyses L Ca2+ signalling in cardiovascular disease: the role of the plasma membrane calcium pumps Sci China Life Sci 2011 54 8 691 698 10.1007/s11427-011-4199-1 21786192

[B2] Nabel EG Cardiovascular disease N Engl J Med 2003 349 1 60 72 10.1056/NEJMra035098 12840094

[B3] Hansson GK Robertson AK Soderberg-Naucler C Inflammation and atherosclerosis Annu Rev Pathol 2006 1 297 329 10.1146/annurev.pathol.1.110304.100100 18039117

[B4] Singh SS Kang PM Mechanisms and inhibitors of apoptosis in cardiovascular diseases Curr Pharm Des 2011 17 18 1783 1793 10.2174/138161211796390994 21631422

[B5] Kim GT Chun YS Park JW Kim MS Role of apoptosis-inducing factor in myocardial cell death by ischemia-reperfusion Biochem Biophys Res Commun 2003 309 3 619 624 10.1016/j.bbrc.2003.08.045 12963035

[B6] Keith M Geranmayegan A Sole MJ Kurian R Robinson A Omran AS Jeejeebhoy KN Increased oxidative stress in patients with congestive heart failure J Am Coll Cardiol 1998 31 6 1352 1356 10.1016/S0735-1097(98)00101-6 9581732

[B7] Cheng TJ Chuu JJ Chang CY Tsai WC Chen KJ Guo HR Atherosclerosis induced by arsenic in drinking water in rats through altering lipid metabolism Toxicol Appl Pharmacol 2011 256 2 146 153 10.1016/j.taap.2011.08.001 21851829

[B8] Plump AS Lum PY Genomics and cardiovascular drug development J Am Coll Cardiol 2009 53 13 1089 1100 10.1016/j.jacc.2008.11.050 19324252

[B9] Frazier L Johnson RL Sparks E Genomics and cardiovascular disease J Nurs Scholarsh 2005 37 4 315 321 10.1111/j.1547-5069.2005.00055.x 16396403

[B10] Mayr M Zhang J Greene AS Gutterman D Perloff J Ping P Proteomics-based development of biomarkers in cardiovascular disease: mechanistic, clinical, and therapeutic insights Mol Cell Proteomics 2006 5 10 1853 1864 10.1074/mcp.R600007-MCP200 16733263

[B11] Tenenbaum A Fisman EZ Boyko V Benderly M Tanne D Haim M Matas Z Motro M Behar S Attenuation of progression of insulin resistance in patients with coronary artery disease by bezafibrate Arch Intern Med 2006 166 7 737 741 10.1001/archinte.166.7.737 16606809

[B12] Nielsen SE Sugaya T Tarnow L Lajer M Schjoedt KJ Astrup AS Baba T Parving HH Rossing P Tubular and glomerular injury in diabetes and the impact of ACE inhibition Diabetes Care 2009 32 9 1684 1688 10.2337/dc09-0429 19502542 PMC2732168

[B13] Voors AA van Geel PP Oosterga M Buikema H van Veldhuisen DJ van Gilst WH Vascular effects of quinapril completely depend on ACE insertion/deletion polymorphism J Renin Angiotensin Aldosterone Syst 2004 5 3 130 134 10.3317/jraas.2004.029 15526248

[B14] Mukhtar RY Reckless JP Statin-induced myositis: a commonly encountered or rare side effect? Curr Opin Lipidol 2005 16 6 640 647 16276242 10.1097/01.mol.0000188414.90528.71

[B15] Lau WC Gurbel PA Antiplatelet drug resistance and drug-drug interactions: Role of cytochrome P450 3A4 Pharm Res 2006 23 12 2691 2708 10.1007/s11095-006-9084-4 17061171

[B16] PRC SPCot Pharmacopoeia of People's Republic of China, 2005 2005 Beijing: Chemical Industry Press

[B17] Li X Xu X Wang J Yu H Wang X Yang H Xu H Tang S Li Y Yang L Huang L Wang Y Yang S A system-level investigation into the mechanisms of Chinese Traditional Medicine: Compound Danshen Formula for cardiovascular disease treatment PLoS One 2012 7 9 e43918 10.1371/journal.pone.0043918 22962593 PMC3433480

[B18] Lim S Yoon JW Kang SM Choi SH Cho BJ Kim M Park HS Cho HJ Shin H Kim YB Kim HS Jang HC Park KS EGb761, a Ginkgo biloba extract, is effective against atherosclerosis in vitro, and in a rat model of type 2 diabetes PLoS One 2011 6 6 e20301 10.1371/journal.pone.0020301 21655098 PMC3107221

[B19] Cheng TO Cardiovascular effects of Danshen Int J Cardiol 2007 121 1 9 22 10.1016/j.ijcard.2007.01.004 17363091

[B20] Kudolo GB Wang W Barrientos J Elrod R Blodgett J The ingestion of Ginkgo biloba extract (EGb 761) inhibits arachidonic acid-mediated platelet aggregation and thromboxane B2 production in healthy volunteers J Herb Pharmacother 2004 4 4 13 26 10.1080/J157v04n04_02 15927922

[B21] Newman DJ Cragg GM Natural products as sources of new drugs over the 30 years from 1981 to 2010 J Nat Prod 2012 75 3 311 335 10.1021/np200906s 22316239 PMC3721181

[B22] Sakure S Negi VD Mitra SK Nandakumar KS Chakravortty D Vaccine with herbal adjuvant--a better cocktail to combat the infection Vaccine 2008 26 27-28 3387 3388 10.1016/j.vaccine.2008.01.060 18502004

[B23] Song X Hu S Adjuvant activities of saponins from traditional Chinese medicinal herbs Vaccine 2009 27 36 4883 4890 10.1016/j.vaccine.2009.06.033 19559122

[B24] Wishart DS Knox C Guo AC Cheng D Shrivastava S Tzur D Gautam B Hassanali M DrugBank: a knowledgebase for drugs, drug actions and drug targets Nucleic Acids Res 2008 36 Database D901 906 18048412 10.1093/nar/gkm958 PMC2238889

[B25] Ye H Ye L Kang H Zhang D Tao L Tang K Liu X Zhu R Liu Q Chen YZ Li Y Cao Z HIT: linking herbal active ingredients to targets Nucleic Acids Res 2011 39 Database D1055 1059 10.1093/nar/gkq1165 21097881 PMC3013727

[B26] Zhu F Shi Z Qin C Tao L Liu X Xu F Zhang L Song Y Zhang J Han B Zhang P Chen Y Therapeutic target database update 2012: a resource for facilitating target-oriented drug discovery Nucleic Acids Res 2012 40 Database D1128 1136 21948793 10.1093/nar/gkr797 PMC3245130

[B27] Magrane M Consortium U UniProt Knowledgebase: a hub of integrated protein data Database (Oxford) 2011 2011 bar009 21447597 10.1093/database/bar009 PMC3070428

[B28] Punta M Coggill PC Eberhardt RY Mistry J Tate J Boursnell C Pang N Forslund K Ceric G Clements J Heger A Holm L Sonnhammer EL Eddy SR Bateman A Finn RD The Pfam protein families database Nucleic Acids Res 2012 40 Database D290 301 22127870 10.1093/nar/gkr1065 PMC3245129

[B29] Gene Ontology Database http://www.geneontology.org/

[B30] Wingender E Dietze P Karas H Knuppel R TRANSFAC: a database on transcription factors and their DNA binding sites Nucleic Acids Res 1996 24 1 238 241 10.1093/nar/24.1.238 8594589 PMC145586

[B31] Ogata H Goto S Sato K Fujibuchi W Bono H Kanehisa M KEGG: Kyoto Encyclopedia of Genes and Genomes Nucleic Acids Res 1999 27 1 29 34 10.1093/nar/27.1.29 9847135 PMC148090

[B32] Zhao J Yang P Li F Tao L Ding H Rui Y Cao Z Zhang W Therapeutic effects of astragaloside IV on myocardial injuries: multi-target identification and network analysis PLoS One 2012 7 9 e44938 10.1371/journal.pone.0044938 23028693 PMC3444501

[B33] Barabasi AL Albert R Emergence of scaling in random networks Science 1999 286 5439 509 512 10.1126/science.286.5439.509 10521342

[B34] Curtis RK Oresic M Vidal-Puig A Pathways to the analysis of microarray data Trends Biotechnol 2005 23 8 429 435 10.1016/j.tibtech.2005.05.011 15950303

[B35] BioCarta Database http://www.biocarta.com/

[B36] Zheng CJ Zhou H Xie B Han LY Yap CW Chen YZ TRMP: a database of therapeutically relevant multiple pathways Bioinformatics 2004 20 14 2236 2241 10.1093/bioinformatics/bth233 15059817

[B37] Gonzalez-Gay MA Gonzalez-Juanatey C Inflammation, endothelial function and atherosclerosis in rheumatoid arthritis Arthritis Res Ther 2012 14 4 122 10.1186/ar3891 22808986 PMC3580546

[B38] Drews J Drug discovery: a historical perspective Science 2000 287 5460 1960 1964 10.1126/science.287.5460.1960 10720314

[B39] Kim NH Kang PM Apoptosis in cardiovascular diseases: mechanism and clinical implications Korean Circ J 2010 40 7 299 305 10.4070/kcj.2010.40.7.299 20664736 PMC2910284

[B40] Seok YM Jin F Shin HM Sung SH Sohn UD Cho JY Kim IK HMC05 attenuates vascular contraction through inhibition of RhoA/Rho-kinase signaling pathway J Ethnopharmacol 2011 133 2 484 489 10.1016/j.jep.2010.10.024 20965238

[B41] Shaw CA Petrik MS Aluminum hydroxide injections lead to motor deficits and motor neuron degeneration J Inorg Biochem 2009 103 11 1555 1562 10.1016/j.jinorgbio.2009.05.019 19740540 PMC2819810

[B42] Rivera E Ekholm Pettersson F Inganas M Paulie S Gronvik KO The Rb1 fraction of ginseng elicits a balanced Th1 and Th2 immune response Vaccine 2005 23 46-47 5411 5419 10.1016/j.vaccine.2005.04.007 16286158

[B43] Yesilada E Bedir E Calis I Takaishi Y Ohmoto Y Effects of triterpene saponins from Astragalus species on in vitro cytokine release J Ethnopharmacol 2005 96 1-2 71 77 10.1016/j.jep.2004.08.036 15588652

[B44] Sun H Ye Y Pan Y Immunological-adjuvant saponins from the roots of Panax notoginseng Chem Biodivers 2005 2 4 510 515 10.1002/cbdv.200590032 17192000

[B45] Nakaya TA Kita M Kuriyama H Iwakura Y Imanishi J Panax ginseng induces production of proinflammatory cytokines via toll-like receptor J Interferon Cytokine Res 2004 24 2 93 100 10.1089/107999004322813336 14980073

[B46] Harvey A Strategies for discovering drugs from previously unexplored natural products Drug Discov Today 2000 5 7 294 300 10.1016/S1359-6446(00)01511-7 10856912

[B47] Strohl WR The role of natural products in a modern drug discovery program Drug Discov Today 2000 5 2 39 41 10.1016/S1359-6446(99)01443-9 10652450

