# Supplementary figures and images for: Target network differences between western drugs and Chinese herbal ingredients in treating cardiovascular disease
Source: BMC Bioinformatics. 2014 Mar 19;15(Suppl 4):S3. doi: 10.1186/1471-2105-15-S4-S3 (PMC4095000; doi:10.1186/1471-2105-15-S4-S3)

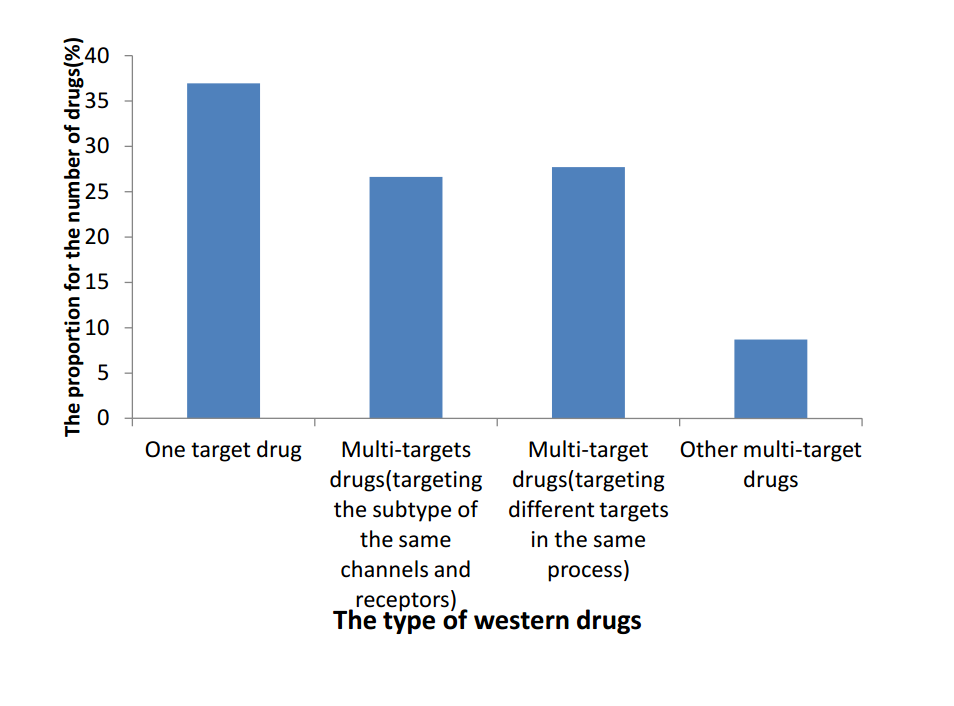

Supplement: Additional file 1 — Figure S1 The distribution of drug type for western drugs. [file 1471-2105-15-S4-S3-S1.png]

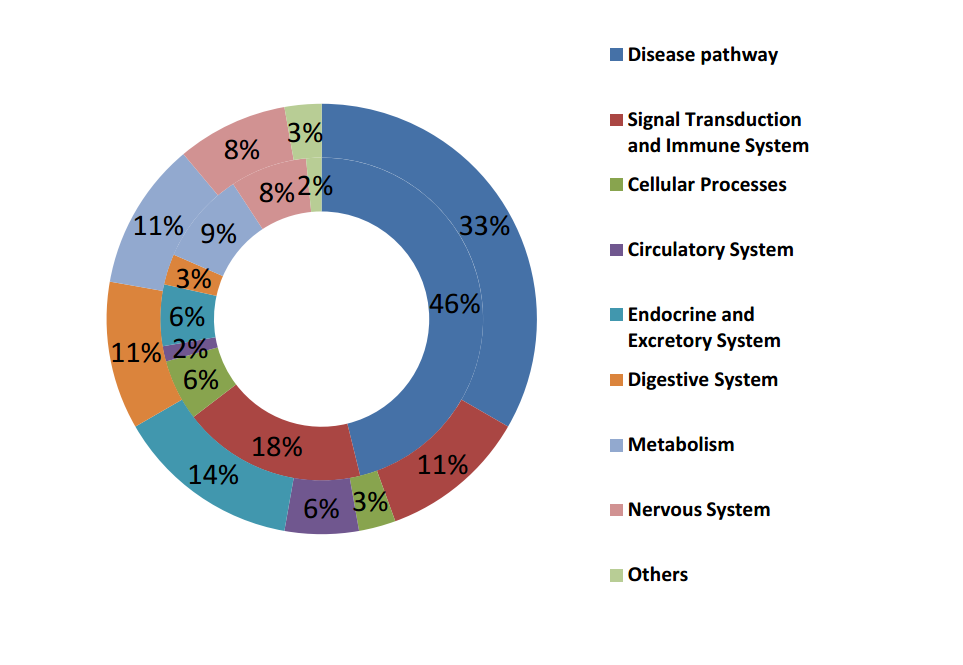

Supplement: Additional file 2 — Figure S2 The proportional distribution of enriched KEGG pathway. [file 1471-2105-15-S4-S3-S2.png]
